# Supplementary material for: The conservation value of freshwater habitats for frog communities of lowland fynbos
Source: PeerJ. 2023 Jun 5;11:e15516. doi: 10.7717/peerj.15516 (PMC10249618; doi:10.7717/peerj.15516)
Supplement: Supplemental Information 1 — Candidate sites in the lowland area of the Agulhas Plain from which final sites were selected for sampling of amphibian and invasive fish communities. Initial categorisations were refined once sites with owner permission were selected. Sites are plotted in Fig. 1. [file peerj-11-15516-s001.docx]

Supplementary Material for:

**Terblanche and Measey: The conservation value of freshwater habitats on frog communities of the Cape lowlands**

Table S1. Candidate sites in the lowland area of the Agulhas Plain from which final sites were selected for sampling of amphibian and invasive fish communities. Initial categorisations were refined once sites with owner permission were selected. Sites are plotted in Figure 1.

| **Number** | **Name** | **Latitude** | **Longitude** | **Category** |
| --- | --- | --- | --- | --- |
| A001 | Pig snout vlei | -34.403397 | 19.375328 | Fynbos stream |
| A002 | Waterfall house pond | -34.404792 | 19.374864 | permanent pond in stream |
| A003 | Christal kloof stream | -34.401153 | 19.399572 | Fynbos permanent stream |
| A004 | No name | -34.409142 | 19.409861 | Fynbos pond |
| A005 | No name | -34.416033 | 19.417989 | Small farm dam |
| A006 | Zilvermyn top dam | -34.414303 | 19.401303 | Medium farm dam |
| A007 | Zilvermynbosch stoor | -34.418178 | 19.404397 | road ditch fynbos |
| A008 | Zilvermynbosch new dam | -34.418394 | 19.403028 | New medium farm dam |
| A009 | Zilvermynbosch bottom dam | -34.419925 | 19.403694 | Medium farm dam |
| A010 | Lochenvarnear river | -34.424639 | 19.41815 | Medium farm dam |
| A011 | Lochenvar dam | -34.423906 | 19.418956 | Small dam |
| A012 | No name | -34.413897 | 19.416975 | medium farm dam |
| A013 | No name | -34.412525 | 19.419308 | medium farm dam |
| A014 | Bosse Clarke top dam | -34.412481 | 19.419958 | medium farm dam |
| A015 | Bosse Clarke kraal dam | -34.415292 | 19.421167 | medium farm dam |
| A016 | Misty Mountain bottom dam | -34.418536 | 19.427422 | medium farm dam |
| A017 | Misty Mountain | -34.417139 | 19.427606 | Small farm dam |
| A018 | Misty Mountain | -34.415933 | 19.427856 | small farm dam |
| A019 | Misty Mountain | -34.41555 | 19.427792 | seepage |
| A020 | Metcalf dam | -34.414183 | 19.439172 | Big irrigation dam |
| A021 | Metcalf | -34.427528 | 19.446303 | Old sand quarry |
| A022 | Metcalf | -34.428111 | 19.440439 | small dam group |
| A023 | Metcalf | -34.43125 | 19.442617 | small dam |
| A024 | Blue Mountain | -34.4275 | 19.461578 | small dam |
| A025 | Arum Lily pond | -34.427994 | 19.460058 | small pond |
| A026 | Blue Mountain | -34.426808 | 19.45915 | small dam |
| A027 | Blue Mountain | -34.426631 | 19.457539 | small dam |
| A028 | Blue Mountain | -34.427886 | 19.457653 | small dam |
| A029 | Blue Mountain bottom dam | -34.430425 | 19.460664 | small dam |
| A030 | Malan | -34.416322 | 19.465533 | Medium farm dam |
| A031 | Malan | -34.416797 | 19.470603 | Medium farm dam |
| A032 | Malan | -34.4179 | 19.470497 | Medium farm dam |
| A034 | Malan | -34.422806 | 19.460561 | Large farm dam |
| A035 | Royal OKE | -34.429272 | 19.465578 | Small pond |
| A036 | Robert Stanford | -34.429797 | 19.464292 | Medium farm dam |
| A037 | Malan | -34.428625 | 19.464886 | medium pond |
| A038 | Malan | -34.427433 | 19.465767 | medium pond |
| A039 | Pepler | -34.429642 | 19.467386 | medium farm dam |
| A040 | Pepler | -34.430314 | 19.467947 | medium farm dam |
| A041 | Pepler | -34.430456 | 19.465356 | medium farm dam |
| A042 | Pepler | -34.430975 | 19.464014 | small dam |
| A043 | Stanford Hills | -34.422708 | 19.474683 | Small dam |
| A044 | Chris de Wit top dam | -34.425994 | 19.476086 | Medium dam |
| A045 | Chris de Witt house dam | -34.425875 | 19.476961 | Big dam |
| A046 | Saun new dam | -34.425789 | 19.484056 | New farm |
| A047 | Saun | -34.423644 | 19.488958 | Small dam |
| A048 | Saun | -34.426839 | 19.489669 | Small dam |
| A049 | Stanford hills | -34.414892 | 19.479919 | Medium farm dam |
| A050 | Stanford hills | -34.413764 | 19.481108 | Medium farm dam |
| A051 | Stanford hills | -34.413111 | 19.482756 | Medium farm dam |
| A052 | Modderrivier West | -34.422031 | 19.512739 | Large pond |
| A053 | Du Plessis | -34.414536 | 19.516886 | Medium pond |
| A054 | Phillipskop | -34.409442 | 19.521747 | Mountain stream pool |
| A055 | Phillipskop house pond | -34.407164 | 19.528219 | Small pond |
| A056 | Burls dam | -34.440008 | 19.476992 | Medium farm dam |
| A057 | Burls dam | -34.439517 | 19.47745 | Medium farm dam |
| A058 | Pelser big dam | -34.434069 | 19.480486 | big farm dam |
| A059 | Pelser medium dam | -34.433006 | 19.481236 | Medium farm dam |
| A060 | Pelser river edge | -34.429375 | 19.477392 | River edge |
| A061 | Kleinrivier Cheese river edge | -34.413594 | 19.531639 | River edge |
| A062 | Stanford valley cottage | -34.413772 | 19.558411 | Small dam |
| A063 | Stanford valley top dam | -34.4171 | 19.562819 | Big farm dam |
| A064 | Stanford valley small dam, | -34.416081 | 19.560047 | Small dam |
| A065 | Stanford Valley long dam | -34.415606 | 19.565061 | Medium farm dam |
| A066 | Stanford Valley middle dam | -34.415203 | 19.562928 | Small farm dam |
| A067 | Stanford Valley Paddock dam | -34.414236 | 19.562611 | Small farm dam |
| A068 | Cheesefactory | -34.418128 | 19.533867 | Big farm dam |
| A068 | Oak grove | -34.421539 | 19.548936 | small pond |
| A069 | A69 Stanford Valley | -34.418908 | 19.55595 | Small pond |
| A070 | Bernt Sabene | -34.424419 | 19.556336 | Small dam |
| A071 | Oak grove | -34.429392 | 19.553997 | Small dam |
| A072 | Oak grove | -34.432686 | 19.553119 | Medium dam |
| A073 | Oak Grove | -34.433183 | 19.549967 | small pond |
| A074 | Oak grove | -34.432008 | 19.546506 | Small dam |
| A075 | Oak grove | -34.428942 | 19.548542 | Small dam |
| A076 | Oak grove | -34.435494 | 19.553417 | Medium dam |
| A077 | Oak grove vleiland | -34.442958 | 19.559219 | Wetland |
| A078 | Eikenhof | -34.4455 | 19.5613 | Medium dam |
| A079 | Eikenhof | -34.445714 | 19.561606 | Medium dam |
| A080 | Haes farm | -34.4441 | 19.562783 | Medium dams |
| A081 | Chrisjan | -34.419919 | 19.536208 | Small dam |
| A082 | Chrisjan | -34.425983 | 19.53185 | Small dam |
| A083 | Chrisjan | -34.427117 | 19.528919 | Smal dam |
| A084 | Chrisjan | -34.4295 | 19.5343 | Smal dam |
| A085 | Chrisjan | -34.431642 | 19.534839 | Smal dam |
| A086 | Modderrivier | -34.432183 | 19.522081 | Smal dam |
| A087 | Chrisjan | -34.436697 | 19.534869 | Smal dam |
| A088 | Modderrivier | -34.437242 | 19.528708 | Smal dam |
| A089 | Modderrivier | -34.436906 | 19.51825 | Small dam |
| A090 | Boschheuwel | -34.453878 | 19.486283 | Small dam |
| A091 | Bosheuwel | -34.445967 | 19.501503 | Small dam |
| A092 | Kleinmodderrivier | -34.443792 | 19.513706 | Medium dam |
| A093 | Modderrivier Andries | -34.445936 | 19.513631 | Small dam |
| A094 | Modderrivier Andries | -34.445667 | 19.510525 | Small dam |
| A095 | Modderrivier Andries | -34.447939 | 19.510661 | Medium dam |
| A096 | Modderrivier Andries nuwedam | -34.441619 | 19.508389 | Large dam |
| A097 | Modderrivier Andries | -34.440283 | 19.508678 | Vlei |
| A098 | Boscheuwel road quarry | -34.4347 | 19.500517 | Old Roadquarry |
| A099 | Hoekvlei | -34.435194 | 19.499736 | Old sandquarry |
| A100 | Modderrivier farmstead | -34.444431 | 19.516892 | Large dam |
| A101 | Modderrivier rooidam | -34.447592 | 19.518456 | Large dam |
| A102 | Modderrivier pompdam | -34.448214 | 19.5232 | medium dam |
| A103 | Modderrivier rivierdamme | -34.450036 | 19.524236 | Medium dam |
| A104 | Modderrivier rivierdamme | -34.451011 | 19.524736 | Medium dam |
| A105 | Modderrivier rivierdamme | -34.452772 | 19.525986 | Medium dam |
| A106 | Flip se paddam | -34.444314 | 19.524964 | Small dam |
| A107 | Crisjan se bosdam | -34.455914 | 19.529919 | Small dam |
| A108 | Walters | -34.460278 | 19.525056 | Small dam |
| A109 | Walters | -34.461372 | 19.526528 | Small dam |
| A109 | Pieters | -34.460175 | 19.537611 | Medium dam |
| A109 | Pieters | -34.464381 | 19.534033 | Medium dam |
| A110 | Brink | -34.470775 | 19.540656 | Large dam |
| A111 | Whitelaw | -34.454922 | 19.546636 | Small dam |
| A112 | Whitelaw | -34.453353 | 19.547797 | Small dam |
| A113 | Vaalvlei dam | -34.445989 | 19.551897 | Large dam |
| A114 | Vaalvlei leidam | -34.446703 | 19.551442 | Large dam |
| A115 | Vaalvlei fontein | -34.446433 | 19.554706 | Spring dam |
| A116 | Kleinrivier King street | -34.437175 | 19.455136 | River edge |
| A117 | Mouton garden | -34.438467 | 19.451758 | Garden pond |
| A118 | Kleinrivier Onderdorp | -34.438467 | 19.451758 | River edge |
| A119 | Meulstroom | -34.436811 | 19.452508 | Stream |
| A120 | Willem appel dam | -34.442931 | 19.453583 | Large dam |
| A121 | Carstens pond | -34.444733 | 19.457219 | Medium pond |
| A122 | Modderrivier West River edge | -34.426544 | 19.511794 | River edge |
| A122 | Fynbos retreat quarry | -34.531642 | 19.477311 | small old quarry |
| A123 | Fynbos retreat dam | -34.528603 | 19.4899 | Large dam |
| A124 | Steynsbos dam | -34.518247 | 19.495064 | Medium pond in fynbos |
| A125 | Near Steynsbos | -34.514711 | 19.4787 | Medium pond in fynbos |
| A126 | Near Fynbos retreat road quarry | -34.525731 | 19.464847 | Fynbos pond |
| A127 | Grootbos Garden Lodge | -34.55 | 19.412883 | Garden pond |
| A128 | Paardeberg | -34.438292 | 19.613406 | Small dam |
| A129 | Paardeberg | -34.44015 | 19.615172 | Small dam |
| A130 | Paardeberg | -34.447806 | 19.603175 | Large dam |
| A131 | Paardeberg | -34.441656 | 19.595969 | Small dam |
| A132 | Paardeberg | -34.441775 | 19.596539 | Small dam |
| A133 | Paardeberg | -34.441858 | 19.589553 | Small dam |
| A134 | Paardeberg | -34.452283 | 19.580539 | Small dam |
| A135 | Beloftebos | -34.451522 | 19.606453 | River pool |
| A136 | Carstens bo | -34.487725 | 19.564311 | Medium dam |
| A138 | Tat | -34.487725 | 19.564311 | River pond |
| A139 | Christiaan | -34.471578 | 19.575336 | Medium dam |
| A140 | Radynsdam | -34.471578 | 19.575336 | Small dam |
| A141 | Karg | -34.480528 | 19.582864 | Big dam |
| A142 | Karg | -34.480528 | 19.582864 | Small dam |
| A143 | Karg | -34.484667 | 19.574675 | Big dam |
| A144 | Karg | -34.484506 | 19.578381 | Medium dam |
| A145 | Mushroom farm | -34.489422 | 19.586769 | Big river pond |
| A146 | Bruinklip | -34.515247 | 19.580678 | Big farb dam |
| A147 | Bruinklip | -34.513478 | 19.576719 | Small farm dam |
| A148 | Bruinklip | -34.508681 | 19.575167 | Small dam |
| A149 | Bruinklip pond in river | -34.508794 | 19.57645 | Pond in river |
| A150 | Bruinklip | -34.518886 | 19.561592 | River pond |
| A152b | Bruinklip dam | -34.515092 | 19.581442 | Large farm dam |
| A152 | Under pivot | -34.520756 | 19.563044 | Dam under pivot |
| A153 | Klein Goedvertrouw | -34.523794 | 19.562486 | Small dam |
| A154 | Goedvertrouw | -34.527211 | 19.536828 | Pond in river |
| A155 | Goedvertrouw | -34.523194 | 19.542994 | Pond in river |
| A156 | Goedvertrouw | -34.518164 | 19.541531 | Small dam |
| A156 | Goedvertrouw | -34.514033 | 19.541967 | Small dam |
| A157 | Goedvertrouw | -34.522117 | 19.546797 | Small dam |
| A158 | Uilkraalriver | -34.542742 | 19.519356 | Pool in river |
| A159 | Kraaienbosch side dam | -34.555717 | 19.49975 | Large dam |
| A160 | Kraaienbosch kloof dam | -34.555686 | 19.485244 | Large dam |
| A161 | Kraaienbosch dam edge | -34.560547 | 19.492208 | Very large irrigation dam |
| A162 | Kraaienbosch dam edge | -34.573661 | 19.479256 | Pool in river under dam |
| A163 | Koos Groenewald pond | -34.565131 | 19.474811 | Small pond |
| A164 | Koos Groenewald dam | -34.563125 | 19.470772 | Medium dam |
| A165 | Uilenkraal ponds | -34.572992 | 19.469689 | pond in river |
| A166 | Uilenes | -34.569508 | 19.467711 | Medium dam |
| A167 | Uilenes middelhuis | -34.568694 | 19.466289 | Large dam |
| A168 | Uilenkhuis Rietdak | -34.567797 | 19.464997 | Large dam |
| A169 | Uilenkhuis pond | -34.569211 | 19.4644 | pond in river |
| A170 | Uilenvlei dam | -34.567033 | 19.462508 | Small dam |
| A171 | Uilenvlei pond | -34.566714 | 19.460828 | pond in river |
| A172 | Uilenvlei dam | -34.565211 | 19.458383 | Medium dam |
| A173 | Corne Swart | -34.561603 | 19.453475 | Large dam |
| A174 | Uilenvlei | -34.562925 | 19.460531 | pond in river |
| A175 | Grootboskloof | -34.556222 | 19.459019 | Medium dam |
| A176 | Flower valley | -34.557097 | 19.464053 | small dam |
| A177 | Flower valley | -34.557458 | 19.464978 | small dam |
| A178 | Flower valley | -34.555786 | 19.465394 | pond |
| A179 | Flower valley | -34.552097 | 19.468747 | Large dam |
| A180 | Flowe valley | -34.548647 | 19.471817 | pond |
| A181 | Grootbos garden lodge | -34.547772 | 19.412528 | Garden pond |
| A182 | Uilkraal pond | -34.582228 | 19.473808 | pond in river |
| A182 | Ayre pond | -34.581011 | 19.462931 | Pond in fynbos |
| A183 | Ayre pond | -34.581828 | 19.456164 | Pond in veld |
| A184 | Venue | -34.578922 | 19.450992 | Seepage |
| A185 | Venue | -34.579667 | 19.448772 | dam |
| A186 | Caravan park dam | -34.594914 | 19.420847 | Dam |
| A187 | No name | -34.587669 | 19.418014 | Dam |
| A188 | Groeneweide | -34.591547 | 19.407939 | pool |
| A189 | Groeneweide | -34.592153 | 19.407942 | pool |
| A190 | Ramsauer | -34.595847 | 19.400092 | Small dam |
| A191 | Ramsauer | -34.594242 | 19.399867 | large dam |
| A192 | Caravan park | -34.595875 | 19.402914 | Roadside ditch |
| A193 | No name | -34.591858 | 19.412678 | small dam |
| A194 | No name | -34.590947 | 19.416903 | small dam |
| A195 | Onder Groeneweide | -34.593047 | 19.409467 | small dam |
| A196 | Franskraal roadquarry | -34.599286 | 19.37705 | previous quarry |

Table S2. Variable Inflation factors (VIF) for environmental and control measures used in partial RDA and NMDS analyses.

|  | **GVIF** | **Df** | **GVIF^(1/(2*Df))** |
| --- | --- | --- | --- |
| Date.visited | 1.3707 | 1 | 1.1708 |
| Latitude | 3.6932 | 1 | 1.9218 |
| Longitude | 1.4953 | 1 | 1.2228 |
| Wetland.type | 14.2988 | 4 | 1.3945 |
| temporary | 4.5803 | 1 | 2.1402 |
| catchment | 3.1053 | 1 | 1.7622 |
| area | 2.0645 | 1 | 1.4368 |
| perimeter | 2.8892 | 1 | 1.6998 |
| pH | 1.8321 | 1 | 1.3535 |
| Conductivity | 3.0521 | 1 | 1.7470 |
| Fish | 2.3111 | 1 | 1.5202 |

Table S2: Site type and the 11 species of amphibians found at 50 lowland sites in the Overberg.

| **Species** | **Fynbos pool** | **Large Dam** | **River Edge** | **Small Dam** | **Temp. Vlei** |
| --- | --- | --- | --- | --- | --- |
| *Amietia fuscigula* | 1 | 11 | 3 | 11 | 1 |
| *Hyperolius horstocki* | 3 | 8 | 0 | 7 | 1 |
| *Tomopterna delalandii* | 2 | 4 | 0 | 4 | 0 |
| *Xenopus laevis* | 3 | 8 | 5 | 15 | 3 |
| *Scelerophys pantherina* | 1 | 4 | 2 | 5 | 0 |
| *Scelerophys capensis* | 1 | 5 | 3 | 3 | 0 |
| *Arthroleptella villiersi* | 1 | 3 | 0 | 2 | 2 |
| *Semnodactylus wealii* | 1 | 1 | 0 | 1 | 1 |
| *Cacosternum australis* | 2 | 8 | 2 | 12 | 5 |
| *Strongylopus bonaespei* | 0 | 0 | 0 | 2 | 2 |
| *Strongylopus grayii* | 3 | 10 | 6 | 12 | 8 |

**Table S3:** Output of the permanova (envfit) for a reduced dataset (36 sites with permanent water only) on the presence or absence of fish in the Overstrand of South Africa. The table is sorted by the coefficient with the species showing least tolerant of fish (highest negative value: *Xenopus laevis*) at the top, and those most tolerant at the bottom (highest positive value: *Scelerophys capensis* and *S. pantherina*).

Analysis of Variance Table

Df 1 34 35

Sums of Squares 0.466 4.756 5.222

Mean Squares 0.466 0.140

F-Model 3.33

R^2^ 0.0892 0.9108 1.0000

Pr(>F) 0.022*

| Species | **Intercept** | **Coefficient** |
| --- | --- | --- |
| ***Xenopus laevis*** | **0.6656** | **-0.2040** |
| *Strongylopus grayii* | 0.6990 | -0.0836 |
| *Cacosternum aggestum* | 0.5134 | -0.0518 |
| *Semnodactylus wealii* | 0.0435 | -0.0435 |
| *Strongylopus bonaespei* | 0.0217 | -0.0217 |
| *Amietia fuscigula* | 0.6120 | 0.0033 |
| *Tomopterna delalandii* | 0.2241 | 0.0067 |
| *Arthroleptella villiersii* | 0.1421 | 0.0117 |
| *Hyperolius horstocki* | 0.3829 | 0.0786 |
| ***Scelerophys pantherina*** | **0.3177** | **0.1438** |
| ***Scelerophys capensis*** | **0.3562** | **0.1823** |
